# Joint statement for assessing and managing high blood pressure in children and adolescents: Chapter 1. How to correctly measure blood pressure in children and adolescents

**DOI:** 10.3389/fped.2023.1140357

**Published:** 2023-04-11

**Authors:** Empar Lurbe, Giuseppe Mancia, Javier Calpe, Dorota Drożdż, Serap Erdine, Fernando Fernandez-Aranda, Adamos Hadjipanayis, Peter F. Hoyer, Augustina Jankauskiene, Susana Jiménez-Murcia, Mieczysław Litwin, Artur Mazur, Denes Pall, Tomas Seeman, Manish D. Sinha, Giacomo Simonetti, Stella Stabouli, Elke Wühl

**Affiliations:** ^1^ CIBER Fisiopatología de la Obesidad y Nutrición (CIBEROBN), Instituto de Salud Carlos III, Madrid, Spain; ^2^ Department of Pediatric, Consorcio Hospital General, University of Valencia, Valencia, Spain; ^3^ University of Milano-Bicocca, Milan, Italy; ^4^ Analog Devices, Inc., Paterna, Spain; ^5^ Department of Pediatric Nephrology and Hypertension, Pediatric Institute, Jagiellonian University Medical College, Kraków, Poland; ^6^ Istanbul University-Cerrahpaşa, Cerrahpaşa Faculty of Medicine, Istanbul, Turkey; ^7^ University Hospital of Bellvitge-IDIBELL, Barcelona, Spain; ^8^ Department of Clinical Sciences, University of Barcelona, Barcelona, Spain; ^9^ School of Medicine, European University Cyprus, Nicosia, Cyprus; ^10^ Department of Paediatrics, Larnaca General Hospital, Larnaca, Cyprus; ^11^ Department of Pediatrics II, University Hospital Essen, Essen, Germany; ^12^ Pediatric Center, Institute of Clinical Medicine, Vilnius University, Vilnius, Lithuania; ^13^ Department of Nephrology, Kidney Transplantation and Hypertension, The Children's Memorial Health Institute, Warsaw, Poland; ^14^ Institute of Medical Sciences, Medical College, Rzeszów University, Rzeszow, Poland; ^15^ Department of Medical Clinical Pharmacology, University of Debrecen, Debrecen, Hungary; ^16^ Department of Medicine, University of Debrecen, Debrecen, Hungary; ^17^ Division of Pediatric Nephrology, University Children’s Hospital, Charles University, Prague, Czechia; ^18^ Department of Pediatrics, University Hospital Ostrava, Ostrava, Czechia; ^19^ Department of Paediatric Nephrology, Evelina London Children's Hospital, Guy's and St Thomas’ NHS Foundation Trust, London, United Kingdom; ^20^ Institute of Pediatrics of Southern Switzerland, Ente Ospedaliero Cantonale (EOC), Bellinzona, Switzerland; ^21^ 1st Department of Pediatrics, Aristotle University of Thessaloniki, Hippokratio General Hospital of Thessaloniki, Thessaloniki, Greece; ^22^ Division of Pediatric Nephrology, Center for Pediatrics and Adolescent Medicine, Heidelberg University Hospital, Heidelberg, Germany

**Keywords:** adolescents, blood pressure, children, hypertension, monitoring

## Abstract

The joint statement is a synergistic action between HyperChildNET and the European Academy of Pediatrics about the diagnosis and management of hypertension in youth, based on the European Society of Hypertension Guidelines published in 2016 with the aim to improve its implementation. The first and most important requirement for the diagnosis and management of hypertension is an accurate measurement of office blood pressure that is currently recommended for screening, diagnosis, and management of high blood pressure in children and adolescents. Blood pressure levels should be screened in all children starting from the age of 3 years. In those children with risk factors for high blood pressure, it should be measured at each medical visit and may start before the age of 3 years. Twenty-four-hour ambulatory blood pressure monitoring is increasingly recognized as an important source of information as it can detect alterations in circadian and short-term blood pressure variations and identify specific phenotypes such as nocturnal hypertension or non-dipping pattern, morning blood pressure surge, white coat and masked hypertension with prognostic significance. At present, home BP measurements are generally regarded as useful and complementary to office and 24-h ambulatory blood pressure for the evaluation of the effectiveness and safety of antihypertensive treatment and furthermore remains more accessible in primary care than 24-h ambulatory blood pressure. A grading system of the clinical evidence is included.

## Introduction

The joint statement is a synergistic action between HyperChildNET and the European Academy of Pediatrics about the diagnosis and management of hypertension in youth, based on the European Society of Hypertension Guidelines published in 2016 with the aim to improve its implementation. The grading system of the clinical evidence reads as follows:

A. Recommendations are based on randomized trials (or systematic reviews of trials) with high levels of internal validity and statistical precision, provided that the trial results can be directly applied to the patients because of similar clinical characteristics and outcomes have clinical relevance.
B. Recommendations are based on randomized trials, systematic reviews, or prespecified subgroup analyses that have lower levels of precision, or need to extrapolate from studies in different populations or using validated intermediate/surrogate outcomes.
C. Recommendations are based on trials that have lower levels of internal validity and/or precision, trials for which non validated surrogate outcomes were used, or results from observational studies.
D. Recommendations are based on expert opinion alone.

The first and most important requirement for the diagnosis and management of hypertension (HTN) is an accurate measurement of office blood pressure (BP), because in adults, studies on HTN as a cardiovascular risk factor and randomized trials on the beneficial effects of antihypertensive treatment have both been obtained *via* office BP measurements (1). However, although conventional office BP measurement represents the standard BP measurement method, in adults out-of-office BP (24-h ambulatory and home BP) has been shown to offer additional advantages, such as a more accurate identification of the BP phenotype, a more accurate prognostic value and a greater BP reproducibility (2–4). Some of these advantages have been confirmed in children, for which reason multiple measurements away from the office setting are increasingly recommended as a useful addition to office measurement in HTN guidelines for adults as well as for children (5).

## Blood pressure measurements

### Office blood pressure

#### Essential information

**Figure 1:**
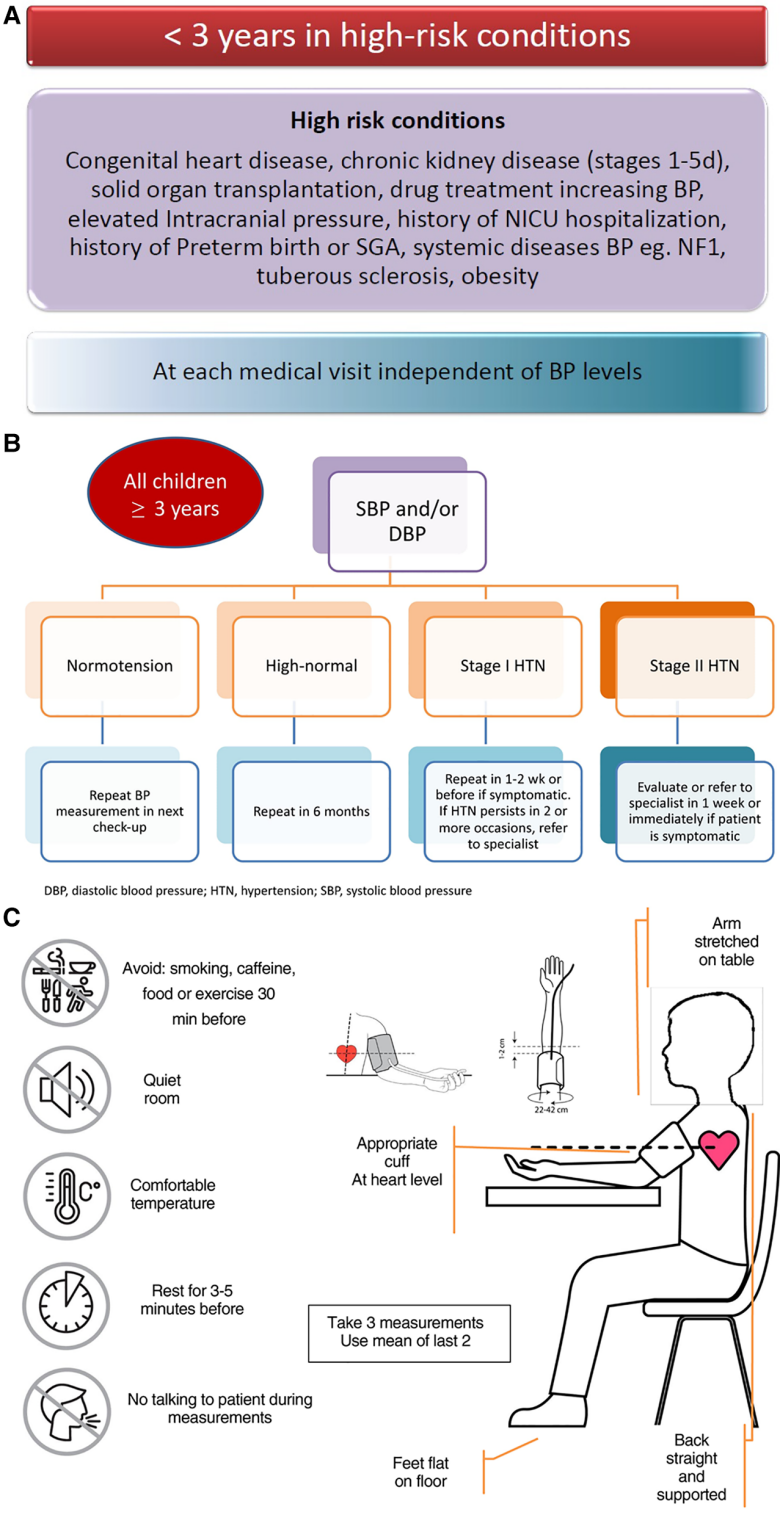
(A-C) Algorithm for frequency of office BP measurement and methodology.

Office BP measurement is currently recommended for screening, diagnosis, and management of high BP in children and adolescents. In cohort studies, high office BP in childhood tracks through adulthood and associates with the presence of subclinical cardiovascular damage (6–8). BP levels should be screened in all children starting from the age of 3 years at well-child visits (Figure 1A). In case of children with risk factors for high BP, office BP should be measured at each medical visit and may start before the age of 3 years (Figure 1B) (9–12). Several studies have shown high variability and low reproducibility of office BP measurements during childhood and adolescence (13). Thus, for the diagnosis of HTN, office BP should be measured on at least 3 different occasions with intervals between visits depending on individual child's BP elevation (10–12) (Figure 1C).

#### Present challenges

Although office BP has been the basis for the management of HTN in children and adolescents some important challenges remain to be solved:

• *Lack of reference values*. Very limited reference data are available with oscillometric devices (14). The largest normative BP database (National High Blood Pressure Education Program or NHBPEP) is based on auscultatory measurements (15–17). Discrepancies between oscillometric BP monitors and auscultatory-based devices may occur due to observer error with auscultation or to worse performance of oscillometric devices (10). Oscillometric devices may provide significantly higher systolic BP values. Although, the overestimation of systolic BP is less when validated devices for children are used, the difference is still significant (18).
• *Validated devices and appropriate cuff sizes* to fit the child´s arm circumference especially for neonates and infants are limited (19, 20).
• *Goals of Treatment*. There are no validated office BP thresholds for treatment based on protection against cardiovascular or renal hard outcomes. In children with primary HTN the recommended office BP target for treatment is a BP < 95th percentile, although BP values < 90th percentile may be preferable because of the increasing evidence from cohort and multicenter studies that cardiovascular risk is increased at office BP levels >90th percentile (6–8, 21). The recommended BP targets in children with chronic kidney disease (CKD) are <75th percentile if there is no proteinuria and <50th percentile with proteinuria. These targets are based on extrapolation of the results of the ESCAPE trial, a multicentre randomized controlled trial that used ambulatory blood pressure monitoring (ABPM) targets to evaluate the effect of strict BP control on CKD progression (10, 22).
• *Ambiguity regarding office BP interpretation and management decisions*. Differences in guidelines among various societies, as well as gaps in knowledge lead to ambiguity regarding office BP interpretation and management decisions. A more protocolized approach and further insight into the reasons for variation in physicianś interpretations could help standardize practice (10–12).
• *Advances in the ability to identify, evaluate, and care for neonates with HTN*. Important gaps need to be addressed to improve the management of neonatal HTN. While no large studies exist, published data of BP percentiles for neonates are available (15, 23–25).

In the following materials we present advantages and limitations of office blood pressure (Table 1), methodology for office blood pressure measurement (Box 1), recommendations for high blood pressure screening at the office (Table 2), common mistakes during office blood pressure measurement (Table 3) and Interpretation of office blood pressure (Table 4).

**Table 1:** Advantages and limitations of office blood pressure.

Advantages	Limitations
• Wide availability in all resource settings • Low cost • Easy to measure • Predictive of future hypertension based on cohort studies	• Observer bias • Low number of measurements • Low reproducibility of measurements • White coat effect • Low availability of validated monitors and appropriate cuff sizes

**Table 2:** Recommendations for high blood pressure screening at the office.

	Grade*
• **Auscultatory or validated oscillometric** devices can be used for office BP measurement in children and adolescents	B
• **Office BP levels should be screened** in all children, starting from the age of 3 years or earlier in children with risk factors for high BP	B
• **For the diagnosis** of hypertension office BP should be elevated on at least 3 different visits	C

*Clinical evidence.

**Table 3:** Common mistakes during office blood pressure measurement.

Methodology	Equipment
• Single measurement • Noisy, cold room • Talking or crying • Neither arm or back support • Hanging/Crossing legs • Measurement over clothes • Diagnosis of high BP by measuring BP on legs or calves, instead of on the right arm	• Small and large cuffs • Non-validated devices • Non-calibrated devices • Cuff and device from different manufacturers • Wrist or forearm devices

**Table 4:** Interpretation of office blood pressure.

Definition of HTN		
Age	BP Category	HTN Threshold
**<16 years**	Normal BP	<90^th^ percentile^a^
	High Normal BP	90^th^ to < 95^th^ percentile^a^
	Stage 1 Hypertension	≥95^th^ to < 99^th^ percentile + 5 mmHg^a^
	Stage 2 Hypertension	≥99^th^ percentile +5 mmHg^a^
	Isolated Systolic Hypertension	SBP ≥95^th^ and DBP < 90^th^ percentile^a^
**≥16 years**	Normal BP	<130/85 mmHg
	High Normal BP	130–139/85–89 mmHg
	Stage 1 Hypertension	140–159/90–99 mmHg
	Stage 2 Hypertension	160–179/100–109 mmHg
	Stage 3 Hypertension	≥180/110 mmHg
	Isolated Systolic Hypertension	SBP ≥140 and DBP <90 mmHg

BP, blood pressure; SBP, systolic blood pressure; DBP, diastolic blood pressure; HTN, hypertension. Isolated systolic hypertension is graded 1, 2, or 3 according to the systolic BP values in the ranges indicated. The interpretation of office blood pressure thresholds is based on the ESH Guidelines (10). It is very well known that there are discrepancies about the definition of hypertension among the European, American and Canadian Guidelines in children (10–12).

^a^ A calculator for office BP classification is provided by HyperChildNET (www.hyperchildnet.eu) under the link https://hyperchildnet.eu/blood-pressure-calculator/.

**BOX 1:** Methodology for office blood pressure measurement.

	***NEONATES OR INFANTS***	***CHILDREN***
***BASIC RECOMMENDATIONS***
• Perform office BP measurement in a quiet room with a comfortable room temperature.	**X**	**X**
• Feeding and sucking during measurement should be avoided.	**X**	
• Perform office BP measurements a reasonable time after medical procedures.	**X**	
• Patients should avoid smoking, caffeine, eating or engagement in exercise 30 min before the measurement.		**X**
• Patients should remain seated and relaxed for 3–5 min before BP measurement.		**X**
• Patients should not talk during or between BP measurements.		**X**
***PROCEDURE***
• The neonate and infant should be resting in prone or supine position.	**X**	
• Patients should be seated with back supported, legs uncrossed, feet flat on the floor.		**X**
• Measure BP on the right arm as indicated in reference tables.	**X**	**X**
• The arm should be bare of clothing and supported at heart level as denoted by the mid-sternal level.	**X**	**X**
• Use auscultatory devices or electronic oscillometric upper-arm cuff devices validated for the patients age according to an established protocol. Currently validated devices for children and adolescents are listed in the HyperChildNET (www.hyperchildnet.eu).	**X**	**X**
• Use appropriate cuff sizes according to the patient's mid-arm circumference. The cuff bladder length needs to cover 80%–100% of the arm circumference and the width 40% of the mid-arm circumference.	**X**	**X**
• Use Korotkoff sound I and V to define systolic and diastolic BP, respectively, in case of auscultatory measurements. Use Korotkoff IV in children where Korotkoff V is zero.	**X**	**X**
• Measure BP three times with an interval of 1–3 min between measurements and calculate the average of the last two values to determine the BP level.	**X**	**X**
• If at first visit there is a high BP, measure BP in both arms and legs to detect possible differences.	**X**	**X**
• If hypertension is detected by the oscillometric method, it needs to be confirmed using the auscultatory method.	**X**	**X**
***INTERPRETATION***
• Evaluate the average office BP using the 95th BP percentile according to BP curves from the N*Η*BPEP reference BP tables or BP percentile data for neonates.	**X**	
• Use the N*Η*BPEP reference BP tables according to age, height and sex adopted by the ESH 2016 guidelines if a child is <16 years old, use the adult thresholds if an adolescent is ≥16 years old. A calculator for office BP classification is provided by HyperChildNET (www.hyperchildnet.eu) under https://hyperchildnet.eu/blood-pressure-calculator/.	**X**	

## Out of office blood pressure

### Twenty-four hour ambulatory blood pressure monitoring

#### Essential information

Twenty-four-hour (24-h) ABPM is increasingly recognized as an important source of information and is regarded as an established tool for the diagnosis of HTN in children and adolescents in several guidelines (10–12, 26). Reproducibility of BP measurement is of fundamental importance for an accurate identification of an individual BP level in absence and during treatment and 24-h mean BP values are indisputably more reproducible than office BP values. Limitations of office BP measurements such as within visit BP variability, the white coat effect, and the observer bias can be avoided by ABPM (10–12, 26, 27). ABPM can detect alterations in circadian and short-term BP variations and identify specific phenotypes such as nocturnal HTN or non-dipping pattern, morning BP surge, white coat and masked HTN (10–12, 26–28) with prognostic significance. In adults, ABPM shows a closer association with HTN outcomes than office BP, and the joint use of the two BP values increases the predictive ability for cardiovascular outcomes (4, 10, 11).

#### Present challenges

Although use of 24-h ABPM in children is now widespread, some important challenges remain to be solved:

• *Lack of reference values for the non-European White Caucasian population*. The most widely used data were published by Wühl and co-workers (29) as age and sex, and height and sex specific reference values from 5 years of age or 120 cm height. As for office BP, the 95th 24-h mean systolic and diastolic BP percentiles are used as HTN thresholds in children and younger adolescents. However, whether to use percentiles or fixed cut-off BP values to diagnose HTN in adolescents is a matter of debate (30, 31). Currently, the 95th percentile is used as a HTN threshold as long as the values are inferior to the accepted criteria for adults (10). This avoids the paradoxical situation in which an adolescent may meet criteria for HTN set by adult guidelines but not by paediatric guidelines. Considering the intraindividual differences between office and ABPM, it is inaccurate to derive reference values for ambulatory BP by extrapolation from office BP values (27).
• *Significance and persistence of white coat hypertension (WCH) and masked hypertension (MH)*. The clinical relevance of WCH and MH in children and adolescents is a matter of debate because: (a) in young people studies on the relationship of these conditions with clinical outcomes are unfeasible (b) whether WCH and MH are associated with more frequent or severe HTN-mediated organ damage (HMOD) has been addressed in only few studies (32, 33) and (c) persistence of these conditions has not been well analysed in prospective studies. Additional difficulties derive from physiological changes of BP due to growth, maturation or pathological changes (34).
• *Validated devices and cuff size* to fit the individuaĺs arm circumference according to the device instructions (19).
• *Goals of Treatment*. There is an ongoing debate on which should be the BP targets for treatment in children younger than 16 years. In children with primary HTN without HMOD, achievement of BP values < 95th percentile may be acceptable and has the advantage of aligning with the cut-off for diagnosis of HTN. In the presence of HMOD or secondary HTN, BP values < 90th percentile are probably preferable. Children with CKD, without proteinuria, should be targeted to a 24-h MAP <75^th^ while in children with CKD and proteinuria, the 24-h BP target should be <50^th^ percentile (10, 22).
• *Clinical trials*. There is a strong need for clinical trials on 24-h ABPM, to facilitate the assessment of efficacy of antihypertensive treatment strategies and their impact on daily life BP, BP phenotypes and BP variability in children. In clinical practice 24-h ABPM increases the accuracy of HTN diagnosis in children and improves the power of clinical trials in pediatric HTN (10, 35).
• *Ambiguity regarding 24-h ABPM interpretation and management decisions*. Differences in guidelines among various societies, as well as gaps in knowledge lead to ambiguity regarding 24-h ABPM interpretation and management decisions. A more protocolized approach and further insight into the reasons for variation in physicianś interpretations could help standardise practice (10–12, 36).

In the following materials we present indications for 24-h ambulatory blood pressure monitoring (Table 5), advantages and limitations of 24-h ambulatory blood pressure monitoring vs. office blood pressure (Table 6), methodology for 24-h ambulatory blood pressure monitoring (Box 2), interpretation of 24-h ambulatory blood pressure monitoring (Table 7), blood pressure phenotypes according to office and ambulatory blood pressure monitoring (Table 8).

**Table 5:** Indications for 24-h ambulatory blood pressure monitoring.

During the diagnostic process	Grade*
• To confirm hypertension before starting antihypertensive drug treatment in order to avoid treatment of white coat hypertension	B
• To exclude masked hypertension in patients with hypertensive mediated organ damage (LVH, microalbuminuria etc) but normal office BP	B
• In children and adolescents with	
▪ Type 1 and Type 2 diabetes	B
▪ Chronic kidney disease	B
▪ Renal, liver or heart transplant	B
▪ Severe obesity with or without sleep-disordered breathing	C
▪ Repair of aortic coarctation	B
▪ Hypertensive response during the treadmill test	D
• Substantial discrepancy between office BP and home BP	D
**During antihypertensive drug treatment**	
• To evaluate apparent drug-resistant hypertension	B
• To assess BP control in children with target organ damage	C
• To exclude symptoms of hypotension	D
**Clinical trials**	B
**Other clinical conditions**	
• Autonomic dysfunction	B
• Suspicion of catecholamine-secreting tumours	D

* Clinical evidence; LVH, left ventricular hypertrophy; BP, blood pressure.

**Table 6:** Advantages and limitations of 24-h ambulatory blood pressure monitoring vs. office blood pressure.

Advantages	Limitations
• Large number of BP measurements • Better reproducibility of mean 24-h, day, or night-time mean values • Assessment of BP during daily-life activities, and sleep • Avoidance of the white coat effect.	• Low availability of validated monitors and appropriate cuff sizes • Low availability of individual devices at doctor's disposal • Cost of the equipment • Higher cost than office BP measurement • Reimbursement by insurance providers • Time consuming procedure • Sleep disturbance

**Table 7:** Interpretation of 24-h ambulatory blood pressure monitoring.

Definition of HTN		
Age	Time Category	ABPM Thresholds
**<16 years**	24-h average	≥95th percentile^a^
	Daytime (awake) average	≥95th percentile^a^
	Night-time (sleep) average	≥95th percentile^a^
**≥16 years**	24-h average	≥130/80 mmHg
	Daytime (awake) average	≥135/85 mmHg
	Night-time (sleep) average	≥120/70 mmHg
**Circadian variability <16 years and ≥16 years**
Dipper^b^	≥10%	
Non-dipper^b^	<10%	

BP, blood pressure; HTN, hypertension.

^a^ 95th percentile is used as a threshold as long as the values are inferior to the accepted criteria for adults (24-h average 130/80; daytime average 135/85 mmHg; nocturnal average 120/70 mmHg) (29, 37). A calculator for ambulatory blood pressure monitoring classification is provided by *HyperChildNET* (www.hyperchildnet.eu) under the link https://hyperchildnet.eu/ambulatory-calculator/.

^b^ Sleep BP dip compared to awake BP (Systolic BP and/or diastolic BP). Apply using individualś sleeping times or fix intervals (midnight to 6 AM).

**Table 8:** Blood pressure phenotypes according to office and ambulatory blood pressure monitoring.

Age	ABPM category	Office SBP and/or DBP	ABPM average SBP and/or DBP
<16 years	Normal BP	<90th percentile	<95th percentile^a^ 24 h and Daytime and Night-time
	Sustained hypertension	≥95th percentile	≥95th percentile^a^ 24 h or Daytime or Night-time
	White-coat hypertension	≥95th percentile	<95th percentile^a^ 24 h and Daytime and Night-time
	Masked hypertension	<90th percentile	≥95th percentile^a^ 24 h or Daytime or Night-time
≥16 years	Normal BP	<140/90 mmHg	24 h <130/80 mmHg and Daytime <135/85 mmHg and Night-time <120/70 mmHg
	Sustained hypertension	≥140/90 mmHg	24 h ≥130/80 mmHg or Daytime ≥135/85 mmHg or Night-time ≥120/70 mmHg
	White-coat hypertension	≥140/90 mmHg	24 h < 130/80 mmHg and Daytime <135/85 mmHg and Night-time <120/70 mmHg
	Masked hypertension	<140/90 mmHg	24 h ≥ 130/80 mmHg or Daytime ≥135/85 mmHg or Night-time ≥120/70 mmHg

ABPM, ambulatory blood pressure monitoring; SBP, systolic blood pressure; DBP, diastolic blood pressure.

^a^ 95th percentile is used as a threshold as long as the values are inferior to the accepted criteria for adults (24-h average 130/80 mmHg; daytime average 135/85 mmHg; nocturnal average 120/70 mmHg) (10, 29, 37).

Box 2 **Methodology for 24-h ambulatory blood pressure monitoring.**

***BASIC RECOMMENDATIONS***

• Perform ABPM in regular school/high school days.
• Do not engage in vigorous exercise during the recording time.
• Patients should remain still with arm extended and relaxed at each measurement.
• Register sleep time, drug intake and symptoms or problems during the recording.
• Patients should know how to switch off the monitor in case of malfunctioning.

***FITTING THE MONITOR***

• Use an electronic oscillometric upper-arm cuff device validated according to a published study using a validated protocol.
• Use cuff size according to the individual's arm circumference.
• Fit cuff on bare non-dominant arm.
• Centre bladder over the brachial artery.
• Set frequency of measurements every 15–20/20–30 min during day/night. The recommended minimal number of readings is 20 during daytime and 7 during night-time.
• Take a test measurement and compare reading with simultaneous office BP measurement. After application, the ambulatory BP should be compared with resting office BP, using a technique similar to the ABPM one (usually oscillometric). If the difference (average of 3 values) is ±5 mmHg or more, the cuff placement should be adjusted, and the procedure repeated. If the difference persists the ABPM device should be checked for calibration.

***REMOVING THE MONITOR***

• Remove the monitor after 24-h.
• Determine day and night-time periods according to patient´s report or set daytime from 8:00 AM to 8:00 PM and night-time from midnight to 6:00 AM.

ABPM patient diary can be found in the supplementary material.

### Home blood pressure

#### Essential information

Home BP (HBP) allows multiple BP measurements away from the office setting, with the advantage of providing measurements on multiple days at rest and away from a potentially stressful environment. Like ABPM home BP values are more reproducible than the office BP values (10, 37). Based on data collected in adults, other ABPM advantages are likely to hold also for home BP. However, at present no data on Home BP advantages for the diagnosis of HTN are available in children while home BP measurements are generally regarded as useful and complementary to office and 24-h ABPM for the evaluation of the effectiveness and safety of antihypertensive treatment (10, 38). Overall, home BP will remain more accessible in primary care than ABPM.

#### Present challenges

Although studies have shown that in children it is feasible to obtain repeated BP measurements within the family environment, there are some challenges and uncertainties:

• *Lack of reference values*. The most widely used data originate from the relatively small Arsakeion School study which provides percentiles tables according to sex and a height of 120 cm and above (39).
• *The relationship between HBP and preclinical hypertensive mediated organ damage is limited*. Very limited evidence exists on the association of HBP with organ damage in children. Studies comparing this association with the association of HMOD with office and 24-h ABPM measurements are needed (10, 40, 41).
• *The variability of HBP monitoring* might induce anxiety in some children and their parents, resulting in too frequent measurements. This can be avoided by careful training (10, 37).
• *Accuracy of the result BP data can be misreported or unreported by parents, relatives, or children themselves*. This can be avoided by using devices with automated memory (37).
• *The role of HBP in children remains controversial*, mainly due to limited evidence of its clinical utility in the young population (10, 11, 38, 41). Relevant research questions on the practical application of HBPM in children should be addressed in future studies
• *Telemedicine* using automatic devices with data transmission may improve the role of HBP.

In the following materials we present advantages and limitations of home blood pressure (Table 9) and methodology and interpretation for home blood pressure (Box 3).

**Table 9:** Advantages and limitations of home blood pressure.

Advantages	Limitations
• To obtain a large number of BP measurements in multiple days away from the office, in the usual environment of each individual • Better reproducibility than office BP • Widely available at relatively low cost • Method for long-term monitoring of treated hypertensive patients • May improve compliance of the patients on antihypertensive medication	• No information on BP during daily life activities, at school and during sleep • Few automated devices have been validated for use in the paediatric population and available cuff sizes are limited • May induce anxiety in children and parents • Possible selective reporting of BP readings • Interpretation, starting treatment time and treatment changes requires medical advice

Box 3 **Methodology and interpretation for home blood pressure.**

***BASIC RECOMMENDATIONS***

• Perform home BP in a quiet room with a comfortable room temperature.
• Avoid smoking, caffeine, eating or engaging in exercise 30 min before the measurement.
• Subjects should remain seated and relaxed for 3–5 min before the initial measurement.
• Subjects should not talk during or between measurements
• Patients and parents require previous training in BP measurement.

***PROCEDURE***

• Subjects should be seated with back supported by chair, legs uncrossed, and feet flat on the floor.
• Subjects should rest their bare arm on table, mid-arm at heart level.
• Use electronic oscillometric upper-arm cuff device validated according to an established protocol.
• Choose cuff size according to the individual's arm circumference, wrapping it around the bare arm (usually left arm).
• Assess home BP ideally for 7 days but for at least 3 days with at least 12 readings.
• Take duplicate morning and evening measurements (before drug intake if treated) with a 1-minute interval.

***INTERPRETATION***

• Preference of automated report and averaging of readings stored in the device memory (or smartphone). Alternatively, review readings reported in a logbook.
• Discard the first day and calculate the average of all the other readings.
• Average home BP ≥95th percentile for sex and height, as long as the values are inferior to the adult criteria ≥135/85mmHg, indicate home HTN.
• Home BP measurement alone not to be used for treatment decisions in children.

Home blood pressure monitoring diary can be found in the supplementary material. A calculator for home blood pressure classification is provided by HyperChildNET (www.hyperchildnet.eu) under the link https://hyperchildnet.eu/paediatric-home-bp-calculator/.

## Conclusion

The first and most important requirement for the diagnosis and management of hypertension is an accurate measurement of office blood pressure. Out-of-office blood pressure measurements in youth is now widely and increasingly recognized as an important source of information. Twenty-four-hour ambulatory blood pressure is regarded as an established tool for the diagnosis of hypertension in children and adolescents with well-known recommendations. Gaps in knowledge lead to ambiguity regarding interpretation and management decisions with relevant research questions on the practical application. Action is required to be addressed in future studies.

## Supplementary material

DATA SHEET 1

Hyperchildnet ABPM patient diary.

DATA SHEET 2

Hyperchildnet HBPM patient diary.
