# Supplementary material for: Joint statement for assessing and managing high blood pressure in children and adolescents: Chapter 1. How to correctly measure blood pressure in children and adolescents
Source: Front Pediatr. 2023 Apr 11;11:1140357. doi: 10.3389/fped.2023.1140357 (PMC10150446; doi:10.3389/fped.2023.1140357)
Supplement: Supplementary file 1 [file Datasheet1.pdf]

ABPM Patient Diary

Name ……………………………………………

Date of ABPM ………………………………………

Hospital (Country) …………………………

Cuff Size (S,M or L) ………………………………

DOB ……………………………………………..

Arm used (R or L) ………………………………

MONITOR OFFICE BP<br>Time				MONITOR 24h-ABPM<br>Time		
SBP	DBP	HR		SBP	DBP	HR

Please complete the following diary<br>and return it with your monitor					
**Started at:**			**Monitor No:**		
Time	Activity/Symptoms	Time	Activity/Symptoms	Time	Activity/Symptoms
Went to bet at:		Woke up at:		**Ends at:**<br>**SWITCH THE MONITOR OFF**	

WARNINGS

- ☐ Sleep and awake/times
- ☐ Medicine/times
- ☐ Switched the machine off?

Please document when you take your blood pressure medication				
**Name of drug/dosage:**	**DAY 1 – DATE:**		**DAY 2 – DATE:**	
	**AM**	**PM**	**AM**	**PM**
