# Supplementary material for: Joint statement for assessing and managing high blood pressure in children and adolescents: Chapter 1. How to correctly measure blood pressure in children and adolescents
Source: Front Pediatr. 2023 Apr 11;11:1140357. doi: 10.3389/fped.2023.1140357 (PMC10150446; doi:10.3389/fped.2023.1140357)
Supplement: Supplementary file 2 [file Datasheet2.pdf]

HyperChildNET COST Action

HOME BLOOD PRESSURE MONITORING

cost EUROPEAN COOPERATION IN SCIENCE & TECHNOLOGY

NAME: ____

Date of birth: ____/____/____ Device:________________________

			Time	Systolic-Diastolic	(Pulse rate)
**DAY 1** __/__/202_	Morning	1er	__:__	____-____	(_____)
		2nd	__:__	____-____	(_____)
	Evening	1er	__:__	____-____	(_____)
		2nd	__:__	____-____	(_____)

Validated electronic arm-cuff device

Before each office visit:

- 7-day monitoring (at least 3)
- Morning and evening, before drug intake
- After 5 min sitting test
- 2 measurements with 1 min interval

Long-term follow-up:
Duplicate measurement once or twice per week or month

			Time	Systolic-Diastolic	(Pulse rate)
**DAY 2** __/__/202_	Morning	1er	__:__	____-____	(_____)
		2nd	__:__	____-____	(_____)
	Evening	1er	__:__	____-____	(_____)
		2nd	__:__	____-____	(_____)

			Time	Systolic-Diastolic	(Pulse rate)
**DAY 3** __/__/202_	Morning	1er	__:__	____-____	(_____)
		2nd	__:__	____-____	(_____)
	Evening	1er	__:__	____-____	(_____)
		2nd	__:__	____-____	(_____)

			Time	Systolic-Diastolic	(Pulse rate)
**DAY 4** __/__/202_	Morning	1er	__:__	____-____	(_____)
		2nd	__:__	____-____	(_____)
	Evening	1er	__:__	____-____	(_____)
		2nd	__:__	____-____	(_____)

			Time	Systolic-Diastolic	(Pulse rate)
**DAY 5** __/__/202_	Morning	1er	__:__	____-____	(_____)
		2nd	__:__	____-____	(_____)
	Evening	1er	__:__	____-____	(_____)
		2nd	__:__	____-____	(_____)

			Time	Systolic-Diastolic	(Pulse rate)
**DAY 6** __/__/202_	Morning	1er	__:__	____-____	(_____)
		2nd	__:__	____-____	(_____)
	Evening	1er	__:__	____-____	(_____)
		2nd	__:__	____-____	(_____)

			Time	Systolic-Diastolic	(Pulse rate)
**DAY 7** __/__/202_	Morning	1er	__:__	____-____	(_____)
		2nd	__:__	____-____	(_____)
	Evening	1er	__:__	____-____	(_____)
		2nd	__:__	____-____	(_____)

WRITE HERE THE AVERAGE OF ALL READINGS EXCEPT OF DAY 1: ____-____ (____)
